# Molecular evidence reveals thyrotropin intervention enhances the risk of developing radioiodine-refractory differentiated thyroid carcinoma

**DOI:** 10.1186/s12935-022-02484-3

**Published:** 2022-02-03

**Authors:** Hilda Samimi, Vahid Haghpanah

**Affiliations:** 1 Endocrinology and Metabolism Research Center (EMRC), Endocrinology and Metabolism Clinical Sciences Institute, Tehran University of Medical Sciences, Tehran, Iran; 2 Personalized Medicine Research Center, Endocrinology and Metabolism Clinical Sciences Institute, Tehran University of Medical Sciences, Tehran, Iran

**Keywords:** Radioiodine refractory, Differentiated thyroid carcinoma, Thyrotropin, Sodium/iodide symporter, Thyroglobulin

## Abstract

Radioiodine (RAI) is the mainstay of treatment for differentiated thyroid carcinoma (DTC) following total thyroidectomy. Nevertheless, about 5% of patients with DTC are RAI-refractory (RAI-R). Understanding the molecular mechanisms associated with DTC during progression towards RAI-R DTC, including thyroid-stimulating hormone levels, may help to explain the pathophysiology of challenging RAI-R DTC clinical cases.

**Graphical Abstract:**

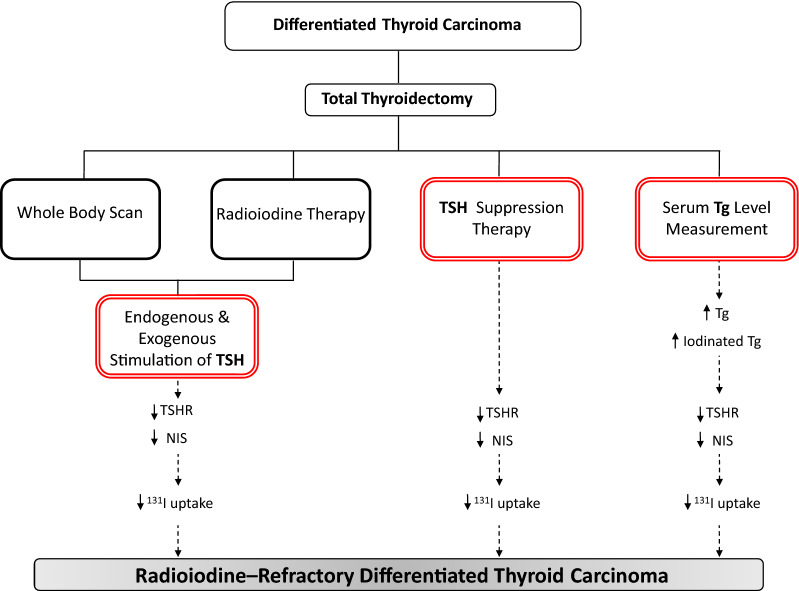

## Background

Thyroid cancer is the most frequent endocrine malignancy and has the highest growing incidence rate between the other kinds of solid tumors in the United States [1]. Differentiated thyroid carcinoma (DTC) accounts for 85–95% of all thyroid cancers [2]. After total thyroidectomy, radioiodine (RAI) ablation followed by long-term suppression of thyroid-stimulating hormone (TSH) through thyroid hormone supplementation is the treatment plan for patients with DTC [3]. RAI is the mainstay of treatment for patients with DTC following total thyroidectomy. However, a small percentage of patients with recurrent DTC have tumors that do not concentrate ^131^I, resulting in RAI resistance, poor prognosis, and a clinical challenge for physicians and patients [4]. Understanding the biological mechanisms associated with DTC progression towards radioiodine-refractory (RAI-R) DTC may therefore be beneficial in providing an effective and appropriate plan for these cases.

In this paper, we aim to hypothesize and discuss the possible molecular association between TSH suppression therapy and thyroid hormone withdrawal before RAI therapy and whole body scan (WBS) as well as degree of iodinated thyroglobulin (Tg) with the increased risk of developing RAI-R DTC, by affecting the regulation of thyroid-stimulating hormone receptor (*TSHR*) and sodium/iodide (Na^+^/I^−^) symporter (*NIS*) expression as the main factor in ^131^I uptake.

### TSH suppression therapy and risk of developing RAI-R DTC

**Fig. 1:**
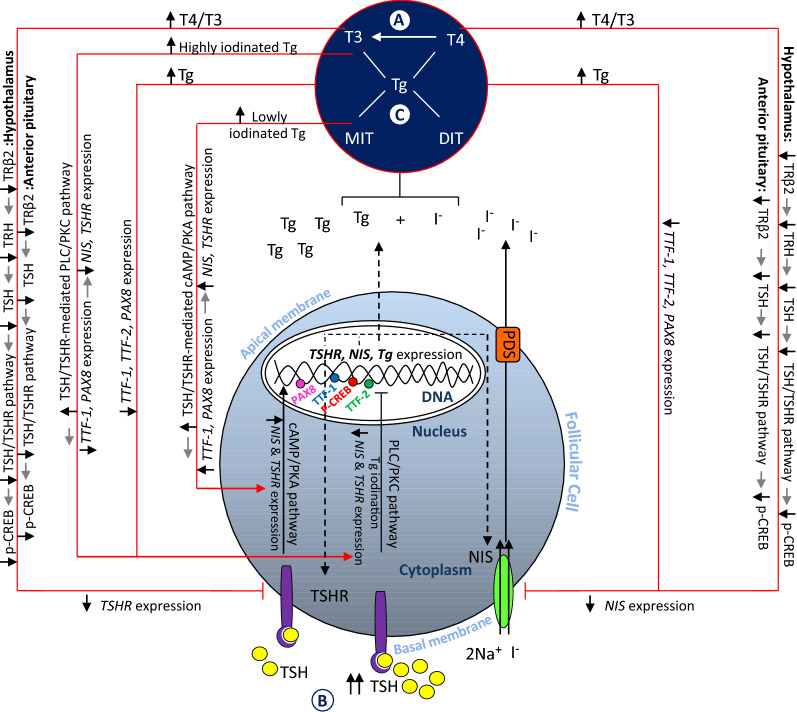
Hypothesized molecular mechanisms of T4/T3 (**A**), TSH (**B**), and Tg (**C**) in developing RAI-R DTC**.** Abbreviations: DIT, di-iodotyrosine; I^−^, iodine; MIT, mono-iodotyrosine; Na^+^, sodium; NIS, sodium iodide symporter; PAX8, paired box 8; p-CREB, phospho-cAMP response element binding protein; PDS, pendrin; PKA, protein kinase A; PKC, protein kinase C; PLC, phospholipase C; T3, triiodothyronine; TRβ2, thyroid hormone receptor beta-2; T4, thyroxine; Tg, thyroglobulin; TRH, thyrotropin releasing hormone; TSH, thyroid-stimulating hormone; TSHR, thyroid-stimulating hormone receptor; TTF-1, thyroid transcription factor 1; TTF-2, thyroid transcription factor 2

Previous studies have shown that TSH-mediated cAMP/protein kinase A (cAMP/PKA) and phospholipase C/protein kinase C (PLC/PKC) signaling pathways are very important for the regulation of *Tg, TSHR*, and *NIS* expression and consequently I^−^ uptake and synthesis of thyroid hormones including triiodothyronine (T3) and thyroxine (T4) [5–9]. The concentration of TSH in the activation of each of these pathways is decisive. Activation of the PLC/PKC signaling pathway requires tenfold higher concentrations of TSH than those needed to activate cAMP/PKA pathway [10–12]. However, D'Arcangelo et al. showed based on the in vitro experiment that the concentrations of TSH from 0.01 to 10 mU/L can also activate this pathway in primary human thyrocytes [13]. Generally, the PLC/PKC signaling pathway inhibits differentiation of thyroid cells such as TSH-stimulated I^−^ uptake ability and cytoplasmic Tg mRNA accumulation [14] and regulates I^−^ efflux and Tg iodination, while the cAMP/PKA pathway increases I^−^ uptake, *Tg* and *NIS* expression, and thyroid differentiation [10, 15] (Fig. 1).

Patients with DTC are given thyroid hormone supplementation after total thyroidectomy in order to suppress TSH. Thyroid hormone supplementation in doses which suppress serum TSH to below the normal levels (0.4–4.2 mU/L) [16] may however cause permanent changes in the expression of thyroid-specific genes. At high T4/T3 concentration, the expression of *NIS* and *TSHR* are likely downregulated due to decreased level of thyroid hormone receptor beta-2 (TRβ2), thyrotropin releasing hormone (TRH), and TSH, via negative feedback loop between the thyroid gland and anterior pituitary and hypothalamus, as well as phospho-cAMP response element binding protein (p-CREB), as an important transcription factor for *NIS* upstream enhancer (NUE) and *TSHR* promoter (Fig. 1A) [7, 17, 18]. Therefore, following long-term TSH suppression therapy, *NIS* and *TSHR* expression may be indirectly reduced by epigenetic modifications due to a decrease in p-CREB, which probably causes permanent changes in the expression of *NIS* and *TSHR* genes to lower than normal levels in thyrocytes. Hence, thyroid hormone supplementation for TSH suppression may impair *NIS* and *TSHR* expression, ^131^I uptake, and response to RAI therapy or WBS.

### Endogenous and exogenous stimulation of TSH and risk of developing RAI-R DTC

Before WBS and RAI therapy, the serum concentration of TSH in patients with DTC should be raised above the normal range, either by thyroid hormone withdrawal or by recombinant human TSH, providing the necessary TSH concentration to stimulate the thyroid cells for sufficient RAI uptake. As mentioned above, TSH induces *TSHR* and *NIS* expression through the cAMP/PKA pathway and increases NIS transfer to the basal membrane of follicular cells. It is important to note that TSH increases TSHR in normal thyrocytes up to a certain limit, while high concentrations of TSH downregulates the expression of *TSHR* [9] and likely *NIS* genes (Fig. 1B). High TSH concentration, even in this short period, can possibly be a factor in decreasing *NIS* and *TSHR* expression at least at the molecular level, ^131^I uptake, and resistance to RAI therapy. Recently, Xiao et al. showed that determining the optimal level of TSH before ^131^I ablation can be clinically useful for achieving a better response to RAI therapy [19]. This clinical evidence can support the current hypothesis.

### Degree of iodinated Tg and risk of developing RAI-R DTC

As mentioned above, TSH/TSHR signaling pathway regulates Tg iodination through PLC/PKC pathway [15]. In addition, TSH-mediated stimulation of thyroid follicular cells induces post-translational modifications of Tg molecules that are necessary for Tg iodination and increased hormonogenic potential of Tg [20]. Evidence has shown that the degree of Tg iodination in the follicular lumen can affect *NIS* [5, 6] and probably *TSHR* expression through activation of cAMP/PKA or PLC/PKC signaling pathways and regulation of thyroid-specific transcription factors, *TTF-1* and *PAX8* (Fig. 1C).

Serum Tg molecules include newly synthesized non-iodinated Tg as well as iodinated Tg containing hormone residues, T3 and/or T4. Studies have shown that circulating Tg molecules may serve as the substrate for production of T3/T4 outside of thyroid gland [21–23]. Druetta et al. demonstrated that Tg released by metastatic DTC mostly, if not exclusively, is non-iodinated glycoprotein [21]. Although, serum Tg level measurement is used as a prognostic biomarker in predicting distant metastasis and recognizing RAI-R DTC, more studies are needed to determine the association between the degree of serum Tg iodination and its regulatory molecular mechanism on *NIS* and *TSHR* gene expression. In addition, studies have indicated that Tg accumulation at the follicular lumen may decrease *NIS* and *TSHR* expression and consequently I^−^ uptake by autoregulating the expression of thyroid-specific transcription factors such as *TTF-1*, *TTF-2*, and *PAX8* [8, 24] (Fig. 1C). Both mentioned mechanisms may occur in RAI-R DTC cases. The reduced expression of *NIS* and *TSHR* may lead to an impaired response to therapeutic and diagnostic RAI uptake.

## Conclusion

Collectively, physiological disturbance of TSH, either when TSH has been suppressed or TSH increased to stimulate thyroid cells, may interfere with the molecular mechanisms referred to in some DTC patients with impaired RAI uptake. Monitoring of TSH levels during follow up could therefore lead to determination of the appropriate TSH cut-off and also TSH suppression or stimulation periods in DTC cases, thereby reducing the likelihood of RAI-R DTC. Moreover, finding the optimal TSH concentration may be more beneficial for patients with *BRAF*^V600E^ and telomerase reverse transcriptase (*TERT*) promoter mutations that are more susceptible to resistance to RAI therapy [25].

Finally, more evidence about RAIR molecular mechanisms, mainly in relation to TSH physiology, can pave the way for identifying patients with increased risk of RAI-R DTC and provide more effective treatment for this challenging paradigm. Alternatively, having genetic and epigenetic patterns of DTC cases could help to predict susceptibility to RAI-R DTC.

## Data Availability

Not applicable.

## Abbreviations

cAMP: Cyclic adenosine monophosphate
DIT: Di-iodotyrosine
DTC: Differentiated thyroid carcinoma
I^−^: Iodine
MIT: Mono-iodotyrosine
Na^+^: Sodium
NIS: Sodium iodide symporter
NUE: NIS upstream enhancer
PAX8: Paired box 8
p-CREB: Phospho-cAMP response element binding protein
PDS: Pendrin
PKA: Protein kinase A
PKC: Protein kinase C
PLC: Phospholipase C
RAI: Radioiodine
RAI-R: Radioiodine-refractory
T3: Triiodothyronine
T4: Thyroxine
TERT: Telomerase reverse transcriptase
Tg: Thyroglobulin
TRH: Thyrotropin releasing hormone
TSH: Thyroid-stimulating hormone
TSHR: Thyroid-stimulating hormone receptor
TSHβ: Thyroid-stimulating hormone beta
TTF-1: Thyroid transcription factor 1
TTF-2: Thyroid transcription factor 2
WBS: Whole body scan
